# Phytolacca Decandra

**Published:** 1856-12

**Authors:** B. Rush Little

**Affiliations:** Keokuk, Iowa


### PHYTOLACCA DECANDRA—A DIURETIC.

BY. B. RUSH LITTLE, M. D.

The Phytolacca Decandra, has, for a long time, enjoyed a high and well merited reputation as a curative agent in certain diseases. Every practitioner in this country, is familiar with it as a remedy much resorted to, in the treatment of chronic rheumatism, syphilis, &c. From considerations of its remedial influence in these diseases, it has been classed by the "authorities" among the alteratives. Observation has shown it to possess also, emetic and cathartic, as well as narcotic properties. But heretofore, it has been valued *in practice* chiefly, as an alterative. It is my purpose in this paper, to prove, that, along with these properties, it possesses *diuretic* powers, and is a most efficient agent in the treatment of dropsy. As far as my information extends, this virtue has not been ascribed to it by any preceding medical writer; and yet so constant and uniform have been the results obtained in my experiments, that it appears singular it should have heretofore escaped observation. I have examined various works on Materia Medica and Therapeutics, as well as Monographus on the subject, and I nowhere find reference had to its diuretic properties, or intimation of its having been used in the treatment of disease, with a view to the production of diuresis.*

---

*I have lately had an opportunity (through the politeness of Prof. Johns,) of referring to "Good's Family Flora," I there find the following passage under the

My experience in the Phytolacca Decandra, I regret to say, has been quite limited; and, doubtless, I will be charged by some of my readers, with having based my conclusions as to its diuretic properties, upon insufficient data. We know it is a well established rule in medical criticism, to reject entirely, or at least receive with hesitancy and reserve, all statements as to the action of medicine, founded upon the evidence of a limited number of facts; and we do not desire to have this rule set aside in the present instance. But the results obtained in the cases reported hereafter, have been so uniform and so well marked, that I feel justified in presenting the subject to the consideration of the profession, for the purpose of having it thoroughly examined. My observations were made whilst engaged in an extensive and miscellaneous country practice, and under circumstances which precluded the ascertaining the particular facts of the cases; such as the changes in the chemical character of the urine, and the exact increase in its quantity which followed the administration of the medicine. Since my change of residence from Pennsylvania to this place, the opportunities I have had for prosecuting my inquiries, have been necessarily limited; but I hope soon to be able to report further experiments made under more favorable circumstances. If my discovery shall be confirmed by subsequent inquiry, we will have in the Phytolacca Decandra, a most invaluable acquisition to our Materia Medica. Being, moreover, an indigenious plant, and growing in all sections of our widely extended country, we will have a cheap as well as efficient remedy always at hand—a consideration of some moment, especially to country practitioners. The importance of ascertaining the medicinal value of our indigenious plants, has not been sufficiently estimated by

---

head of Phytolacca Decandra: "It is a diaphoretic, cathartic and diuretic. These effects probably proceed from the narcotic and anodyne qualities above noticed. When an emetic is exhibited in small doses, diaphoresis generally is the result, especially if combined with an opiate; and frequently emetics, when they pass unchanged from the stomach to the intestines, prove cathartic; such substances, indeed, often show diuretic effects."

It will be seen from the above extract that Mr. Good recognizes the diuretic powers of the Phytolacca Decandra. But from the simple passing notice it receives at his hands, we judge his views as to its diuretic action are merely theoretical inferences, and not the deductions of his experience. Moreover, he does not speak of it as ever having been used in practice with a view to its diuretic properties. His statements are made without allusion whatever to authorities.

the profession generally; and if the publication of this artic] shall give an impulse to inquiries of this kind, we feel assure much will be done for the advancement of practical medicine. With these preliminaries, I will proceed to relate the history of my discovery.

In the fall of 1855 I had several cases of chronic rheumatism under my care, which I treated with the Phytolacca Decandra. In the course of the treatment, my attention was directed to the *diuretic action* of the remedy. As no writer on Materia Medica, with whose works I was acquainted, mentions this as one of the properties of the Phytolacca Decandra, I presumed the diuresis in these cases to be merely coincident and accidental phenomenon, and as having no relation whatever to the remedy under consideration. But the phenomenon here observed was sufficiently prominent to give rise to the inquiry, whether it was not possible that this fact might have escaped the notice of preceding observers; and if the Phytolacca Decandra might not indeed possess diuretic properties? Influenced by this suggestion, I determined to investigate the matter. In the first place, I made inquiry upon this point, of all persons whom I knew to have used the medicine, and uniformly received an affirmative reply. Two suitable cases for experiment, shortly afterward, opportunely presented themselves in the persons of a couple of negroes, who were afflicted with dropsy—the sequella of malarial fever. In these cases, there was ascites, with an anasarcous condition of the lower extremities. I directed these patients to prepare a saturated tincture of the berries, and take one teaspoonful of it three times a day, in connection with 3 or 4 grs. of massa hydrarg., at bed time. (The mercury was prescribed with a view to its alterative influence on the liver, which was evidently in a torpid and engorged condition.) I had not an opportunity of again seeing my patients for five or six days, when I was equally gratified and surprised to find them *entirely relieved.* The ascites as well as the serous effusions in the cellular tissues of the extremities, was altogether removed, and the liver was restored to healthy action. On inquiry as to operation of the medicine, I ascertained it produced, in both cases, most profuse diuresis. This was confirmation sufficient to establish, in my mind, the claims of the Phytolacca Decandra to a place in the Materia Medica, amongst the diuretics. And I should now resort to it in any case in which a diuretic was indicated, with the full assurance of having my expectations realized.

During the following winter, I had but few opportunities of testing its abilities; but in all cases in which I did use it, it gave unequivocal evidence of its diuretic powers.

Owing to my limited experience with the Phytolacca Decandra, I am unable to pronounce positively as to what pathological conditon it is peculiarly applicable; but I consider it indicated in all cases of dropsy, dependent upon congestion of the portal system or torpidity and engorgement of the liver. We have reason to believe it exercises an alterative influence over the liver; this, however, is not well ascertained. But I am by no means willing to limit its applicability to such cases alone; as in the cases of rheumatism above referred to, there was no evidence of any especial derangement of the liver, or congestion of the portal system, and I can see no reason why it should not act as well as in any other forms of dropsy, although I have no other facts upon which to predicate the opinion. I might speculate further on the subject, but prefer to give a simple statement of facts, and leave it to the reflection of the readers of the *Journal*.

KEOKUK, Iowa, Dec., 1856.

